# Patient address parsing via KG-aware contrastive learning and constrained on-prem LLM inference

**DOI:** 10.1038/s41598-026-39348-z

**Published:** 2026-02-09

**Authors:** Jinzhe Li, Xin Pan, Yanchao Jia

**Affiliations:** Information Center, Civil Aviation General Hospital, Chaoyanglu, Beijing, 100123 Beijing China

**Keywords:** Address parsing, Knowledge graph, Contrastive learning, LLM, Engineering, Mathematics and computing

## Abstract

Address parsing seeks to map noisy, abbreviated free-text addresses into standardized hierarchical tuples for large-scale information systems. Existing approaches struggle with semantic and structural ambiguity, hallucination from unconstrained generation, and deployment constraints under privacy and governance requirements. We present AddrKG-LLM, a two-stage framework that combines knowledge-graph (KG)–aware retrieval with schema-restricted large language model (LLM) decoding. First, contrastive learning over multi-view administrative graphs yields node embeddings that retrieve and re-rank a compact Top-K candidate set, thereby bounding the search space while preserving high gold coverage (Recall@K). Second, a candidate-restricted decoder running on-premises produces JSON-compliant outputs, enforcing single-candidate field consistency and alignment with KG priors to improve controllability and policy compliance. Using de-identified real-world records, we evaluate structural consistency via micro-level accuracy ($A_{micro}$) and macro-level accuracy ($A_{macro}$), and assess system properties with Recall@K and latency. Across strong string-matching, sequence-labeling, and generic LLM baselines, AddrKG-LLM delivers consistent gains in $A_{micro}$ and $A_{macro}$ with a favorable Recall@K. The proposed method consists of three components: (i) multi-view graph aggregation, (ii) a hierarchy-aware self-supervised contrastive objective that derives positives/negatives from administrative relations to align textual and graph embeddings, and (iii) candidate-restricted decoding within the KG-derived Top-K set. Overall, coupling KG-aware retrieval with constrained on-prem LLM decoding yields an accurate, controllable, and deployable solution for noisy-address structuring across domains.

## Introduction

Patient address data are key geographic identifiers that support disease transmission modeling, public health decision-making, and the optimal allocation of healthcare resources^1^. High-quality and structured address data also enables efficient service delivery and benefit hospital operations such as patient follow-up and community health interventions^2^. Historically, medical information systems prioritized immediate in-hospital needs over long-term data governance and system integration. Consequently, many records store addresses as free text with heterogeneous expressions, missing fields, and inconsistent formats^3^. The frequent omission of district or street names reduces reliability and downstream applicability^4^. Address records may also contain identifiable elements (e.g., house and building numbers); without effective de-identification, these details create privacy risks. Automated de-identification is therefore required to mask sensitive fields while pre-serving parsing accuracy and utility^2,5^.

Traditional string-matching methods (e.g., edit distance, cosine similarity) work under standardized input but degrade with abbreviations, omissions, word-order variation, and place-name ambiguity be-cause character-level similarity ignores contextual semantics^2^. Natural language processing models such as BERT improve robustness through contextual representation learning and can be deployed with modest inference cost in local medical systems^6,7^. Knowledge graphs (KGs) further provide hierar-chical administrative constraints and field completion by integrating standardized toponyms, historical variants, and administrative divisions, improving alias disambiguation and stability^8,9^.

Recent generative large language models (LLMs) combine semantic understanding with structural text generation and, in open-source variants such as DeepSeek R1, support on-premises inference suita-ble for compliance in healthcare settings^10–13^. However, unconstrained generation can yield halluci-nated content and inconsistent field formatting, which conflicts with the accuracy, stability, and control-lability required for structured address parsing.

To address these issues, we propose AddrKG-LLM, a KG-constrained LLM framework for medical address parsing. In preprocessing, regular-expression rules de-identify inputs by removing building numbers, room numbers, and contact details, and Sentence-BERT encodes dense semantics^14^. We construct an administrative-division KG and train GraphSAGE to capture multilevel geographic de-pendencies. To align text and graph semantics without manual labels, we introduce a self-supervised contrastive objective that derives positive and negative pairs from the KG hierarchy and improves em-bedding consistency. All community-node embeddings are indexed with FAISS, the retrieved Top-k can-didates are injected into restricted prompt templates to form a constrained search space with explicit ge-ographic priors. Inference runs on an on-premises DeepSeek R1 model, generation is restricted to the candidate set, and outputs follow a standardized JSON schema (administrative division, street/township, community). This design reduces hallucination, improves structural consistency and controllability, and supports privacy-preserving deployment. In summary, the main contributions of this paper are as follows:

1. This paper proposes AddrKG-LLM, a two-stage framework for patient address structuring that integrates knowledge graph–enhanced, semantic-constrained retrieval with controlled on-premises LLM inference. By retrieving a KG-bounded Top-K candidate set and decoding strictly within this search space, AddrKG-LLM reduces hallucination, and improves controllability under privacy-preserving, on-premises deployment.

2. This paper introduces a hierarchy-aware self-supervised contrastive learning objective, which automatically constructs positive and negative pairs from administrative divisions encoded in the knowledge graph. The objective aligns textual and graph-based embeddings without manual annotations, thereby improving semantic–structural consistency in retrieval and re-ranking. 3. This paper develops a candidate-restricted decoding mechanism with standardized JSON outputs, which operationalizes constrained generation within the KG-derived Top-K candidate set, enforces sin-gle-candidate field consistency, and mitigates spurious outputs, while supporting privacy-preserving on-premises deployment in sensitive domains.

4. This paper presents extensive experiments on de-identified real-world medical address data, demonstrating consistent improvements over strong baselines in $A_{micro}$ and $A_{macro}$, along with ro-bustness and reproducibility under resource-constrained local deployment conditions.

## Related work

Patient address data governance is a central challenge in medical informatization and intelligent healthcare. The research trajectory has progressed from string-matching algorithms to natural language processing (NLP) techniques, and subsequently to the integration of KGs. Drawing on related studies, this paper categorizes and analyzes the main tasks and methods along this technical trajectory to high-light the novelty of our approach and its value for addressing the problem.

### Applications of string-matching in address parsing

In early research on the governance of unstructured patient address data, traditional string-similarity–based methods were widely adopted. These approaches estimate match quality by computing similarity between strings, targeting spelling errors and formatting differences. Representative techniques include edit distance, cosine similarity, and the Jaro–Winkler distance.

Edit distance is a classic algorithm that measures similarity via insertions, deletions, or substitutions^3^. On standardized data, it can effectively correct minor spelling errors. However, it is sensitive to character order and fails on inputs that are semantically equivalent but reordered^4^. In addition, its computational cost is high on large datasets, making efficiency a key bottleneck.

Cosine similarity represents strings as vectors and measures the cosine of the angle between them to assess textual similarity^7^. Compared with edit distance, it can capture partial overlaps. Nevertheless, its effectiveness depends on the quality of the vector representation, and it can-not handle spelling errors or missing fields. Moreover, cosine similarity lacks semantic modeling, it performs poorly when address parsing requires sensitivity to word order or contextual dependencies^8^.

To strengthen prefix matching, the Jaro–Winkler distance assigns higher weights to shared prefixes, improving discrimination among similar addresses^5^. That said, the improvement remains at the character level and does not resolve semantic ambiguity or missing-field issues, so its applicability is limited.

Overall, string-similarity–based methods have clear advantages for standardized data, minor spelling correction, and simple structural matching. They are easy to implement and suitable for small-scale datasets. However, they suffer from the following limitations^15^: (i) lack of semantic under-standing, making complex or synonymous expressions difficult to handle; (ii) limited capacity to model context and the logical relationships among fields; and (iii) poor adaptability to non-standard inputs, es-pecially when spelling errors and missing fields co-occur. These limitations motivate subsequent research that integrates natural language processing techniques and knowledge graphs.

### Applications of deep learning in address parsing

In recent years, research on applying deep learning to address parsing has advanced steadily. Se-quence-labeling models and pretrained language models have been introduced into address text parsing, significantly improving the accuracy of processing unstructured address data.

Early studies commonly formulated address parsing as a sequence-labeling problem, using recurrent neural networks (RNNs) and their variants to segment address elements into structured fields. A repre-sentative approach is the Bi-LSTM-CRF model: Huang et al.^16^ leveraged bidirectional LSTMs to cap-ture contextual dependencies, while a conditional random field (CRF) explicitly modeled label interac-tions. Ma and Hovy^17^ further incorporated convolutional features, yielding the BiLSTM-CNN-CRF model, which performed strongly across sequence-labeling tasks and improved capture of local context and character-level features.

However, RNN-based architectures (e.g., LSTM, GRU) face vanishing gradients, limited capacity for long-range dependencies, and constrained generalization^18^. With the rise of Transformer-based pre-trained language models (PLMs), BERT^6^ has become central to Chinese address parsing. Unlike fixed word embeddings (e.g., Word2Vec, GloVe), BERT employs a masked-language-modeling objective to learn contextualized token representations, thereby better expressing semantic relations among address elements. Building on BERT, researchers have explored variants to enhance performance and generalization for Chinese address parsing. For example, Zhang et al.^19^ combine BERT contextual modeling with CRF global sequence optimization to achieve precise segmentation of address elements. To mitigate limitations such as semantic ambiguity and domain-specific terminology gaps during pretraining, some studies expand BERT vocabulary, substantially improving recognition of out-of-vocabulary items in address text^20,21^.

In summary, deep learning–based sequence labeling and pretrained language models have become mainstream technical approaches for Chinese address parsing, offering clear advantages in semantic capture and contextual understanding. Nevertheless, these models still entail high architectural complex-ity and substantial training resource consumption.

### Applications of KGs in address parsing

As the advantages of KGs for semantic augmentation and relational reasoning have become salient, they have been increasingly adopted for address parsing, serving as an important instrument for strengthening address data governance^9^. Early KG-based approaches focused on standardization and se-mantic matching. Liu et al.^22^ proposed a graph-based method for Chinese address matching that utilizes a knowledge graph to represent and match address elements. The approach aggregates spatial and hierarchical information from various administrative levels to improve matching accuracy. However, the method is limited by its reliance on predefined matching rules and struggles to handle non-standard address formats and noisy data effectively.

Feng et al.^23^ proposed an entity-alignment interaction model based on Chinese RoBERTa for address matching tasks. The model leverages the RoBERTa pre-trained language model to capture semantic relationships between address components, improving alignment accuracy for complex and noisy data. However, the approach faces challenges in handling address elements with ambiguous semantics and in ensuring robustness across diverse address formats.

Besides, Liu et al.^24^ introduced K-BERT, a model that integrates knowledge graph embeddings with BERT to enhance language representation for tasks involving structured knowledge. The model utilizes knowledge graph information to refine the contextual understanding of words, improving performance on a range of natural language processing tasks. However, K-BERT’s reliance on static knowledge graph embeddings may limit its adaptability to dynamic, real-time knowledge updates.

In summary, KGs strengthen semantic understanding and constrain propagation in address parsing, yet practical deployment remains challenged by high curation and maintenance costs, difficulties in dy-namic updating, and increased model complexity.

## Methods

### AddrKG-LLM framework

**Fig. 1:**
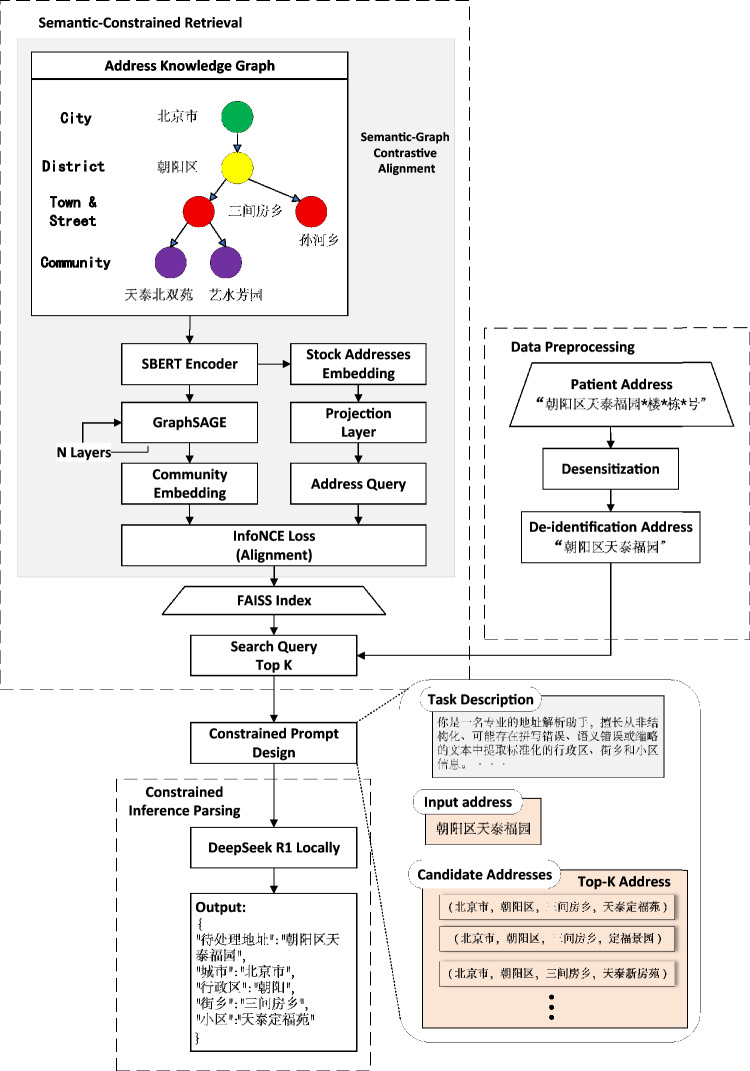
AddrKG-LLM framework.

Figure 1 presents the overall framework of the proposed AddrKG-LLM. The system comprises four core modules:

(i) Data Preprocessing, which performs de-identification with regular expressions (removing build-ing and room numbers and contact information), normalizes tokens and punctuation, and prepares in-puts for downstream encoding.

(ii) Semantic-Constrained Retrieval, which aligns address text to a knowledge-graph–induced em-bedding space using Sentence-BERT and GraphSAGE and retrieves top-k candidate communities via FAISS under administrative constraints.

(iii) Constrained Prompt Design, which instantiates restricted prompt templates with the retrieved candidates and explicit geographic constraints derived from the administrative hierarchy.

(iv) Constrained Inference Parsing, which executes controlled, on-premises inference (e.g., DeepSeek R1), constrains decoding to the candidate set, and returns outputs in a standardized JSON schema city, administrative division, street/township, community.

Collectively, these modules standardize unstructured address inputs and enforce administra-tive-hierarchy–aware semantic consistency, enabling controlled, KG-constrained LLM inference under resource-constrained, on-premises settings.

### Data preprocessing

The data preprocessing module converts unstructured address text provided by patients in the hos-pital master index database into a normalized and standardized form and performs de-identification to remove identifying elements. The processed data serves as high quality input for subsequent semantic encoding and knowledge graph matching.

#### Patient address de-identification and encoding

To ensure privacy compliance and improve downstream matching accuracy, the raw patient address text is processed before modeling. We define a set of regular expressions for identifying fields such as building number, unit number, floor, room number, and mobile phone number:

1 $$\begin{aligned} {\mathcal {R}}=\{\,R_{\text {bracket}},\,R_{\text {num}},\,R_{\text {symbol}},\,\ldots \,\}. \end{aligned}$$

where $R_{\text {bracket}},\ R_{\text {num}},\ R_{\text {symbol}}$ represent removal of parenthetical content, removal of trailing digits with building or room suffixes, and removal of common punctuation, respectively.

For an address *s*, the de-identification function $\operatorname {sanitize}(\cdot )$ is defined as:

2 $$\begin{aligned} \acute{s}=\operatorname {sanitize}(s,{\mathcal {R}}) = s - \bigcup _{r\in {\mathcal {R}}} \operatorname {match}(r,s). \end{aligned}$$

where $\operatorname {match}(r,s)$ represents the set of substrings matched by rule *r*, “−” represents the deletion operation, and $\acute{s}$ represents the de-identified address text.

After de-identification, the processed text $\acute{s}$ is encoded with Sentence-BERT to obtain a *d*-dimensional embedding:

3 $$\begin{aligned} x = f_{\text {SBERT}}(\acute{s}) \in {\mathbb {R}}^{d}. \end{aligned}$$

where $f_{\text {SBERT}}$ represents the Sentence-BERT encoder, *x* represents the address embedding, and *d* represents the embedding dimension. The embedding *x* is then used to compute semantic similarity with knowledge graph node embeddings in subsequent steps.

#### Knowledge graph and node initialization

We constructs the standard address knowledge graph as a directed graph:

4 $$\begin{aligned} G=(V,E). \end{aligned}$$

where *V* represents hierarchical administrative units including city, district, street or township, and community, and *E* represents subordination relations along the administrative hierarchy.

Each node $v\in V$ has an initial embedding $h_v^{(0)}\in {\mathbb {R}}^{d}$. The initial semantic embedding is obtained with Sentence-BERT^14^:

5 $$\begin{aligned} h_v^{(0)}=f_{\text {SBERT}}(t_v)\in {\mathbb {R}}^{d}. \end{aligned}$$

where $t_v$ represents the textual label of node *v*, and *d* represents the embedding dimension.

### Semantic-constrained retrieval

This module is designed to construct an address embedding space that is consistent in semantics and structure, thereby supporting efficient retrieval of standard addresses. In common contrastive learn-ing practice, supervision signals are obtained without manual labels through instance discrimination, data augmentation, or structure-induced neighborhoods. This paper derives such self-supervised signals from the hierarchical relations encoded in the standard address knowledge graph, which enables auto-matic construction of positive and negative pairs and allows label-free training.

#### Graph structural representation learning

Based on the initial representations, this paper adopts GraphSAGE to aggregate neighbor information and capture multilevel administrative relations. The node representation at layer *k* is computed as:

6 $$\begin{aligned} h_v^{(k)}=\sigma \!\left( W_{1}^{(k)}\,h_v^{(k-1)} + W_{2}^{(k)} \cdot \operatorname {MEAN}\!\big (\{\,h_u^{(k-1)} \mid u \in {\mathcal {N}}(v)\,\}\big ) + b \right) . \end{aligned}$$

where ${\mathcal {N}}(v)$, $W_{1}^{(k)}$, $W_{2}^{(k)}$, *b*, and $\sigma (\cdot )$ represent the neighbor set of node *v*, the trainable weight matrices at layer *k*, the bias vector, and the activation function, respectively, and $\operatorname {MEAN}(\cdot )$ represents the mean aggregator. After *L* layers of aggregation, we obtain $h_v^{(L)}$.

#### Semantic projection and alignment

To align the embeddings of composite address text with graph node embeddings, this paper intro-duces a shared projection network:

7 $$\begin{aligned} {\tilde{a}}_u=\phi (a_u),\qquad {\tilde{h}}_v=\phi \!\left( h_v^{(k)}\right) . \end{aligned}$$

where $\phi (\cdot )$ represents a two-layer feed-forward projection shared by both branches, and $a_u=\sum _{\text {district}} h_i^{(0)}$ represents the encoding of the *i*-th cell in the initial graph after concatenating all upper-level administrative districts.

Similarity is computed by cosine similarity:

8 $$\begin{aligned} \operatorname {sim}(i,v)= \frac{{\tilde{a}}_i\cdot {\tilde{h}}_v}{\Vert {\tilde{a}}_i\Vert \,\Vert {\tilde{h}}_v\Vert },\quad v\in V_{\text {comm}}. \end{aligned}$$

### Contrastive learning objective

During training of the graph encoder, only standard addresses from the knowledge graph are used and no manual labels are required. Positive pairs are formed by a composite address ${\tilde{a}}_u$ and its corresponding community embedding ${\tilde{h}}_{v_i}$. Negative samples are other community embeddings within the same mini-batch. The model is optimized with the InfoNCE loss:

9 $$\begin{aligned} {\mathcal {L}} = - \sum _{i=1}^{N} \log \frac{\exp \!\left( \operatorname {sim}({\tilde{a}}_i,{\tilde{h}}_{v_i})/\tau \right) }{\sum _{j=1}^{N}\exp \!\left( \operatorname {sim}({\tilde{a}}_i,{\tilde{h}}_{v_j})/\tau \right) }. \end{aligned}$$

where *N* represents the batch size, and $\tau$ represents the temperature parameter.

#### Retrieval mechanism

After training, all community embeddings $h_v$ are stored in a FAISS index to support approximate nearest-neighbor search. At inference time, a patient address embedding *x* is projected to ${\tilde{x}}$ and the $Top\!-\!K$ most similar communities are retrieved:

10 $$\begin{aligned} C_x = \operatorname {TopK}_{v\in V}\!\big (\operatorname {sim}({\tilde{x}},\,{\tilde{h}}_v)\big ). \end{aligned}$$

where ${\tilde{x}}$ represents the Sentence-BERT encoding of the input address and $C_x$ represents the retrieved candidate set. The candidates are then passed to the downstream restricted-inference module to generate the final standardized address under explicit task constraints.

### Constrained prompt design

**Fig. 2:**
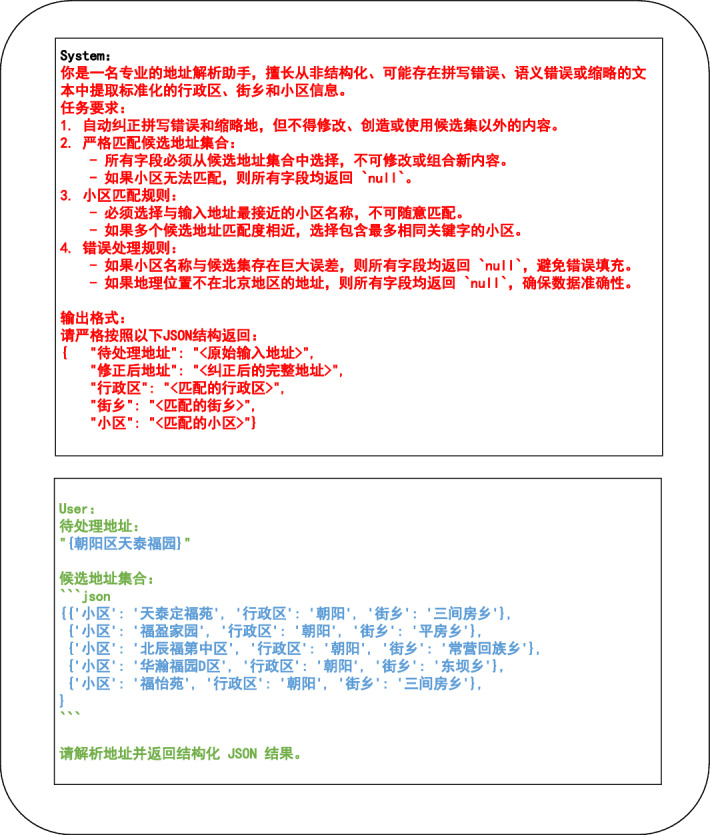
Constrained prompt template.

To control the decoding scope of the language model and mitigate generation drift, this paper designs a constrained prompt anchored to the semantic candidate set, so that generation decisions are conditioned only on the retrieved community candidates. The module takes unstructured address text *x* as input and outputs a prompt string $\operatorname {Prompt}(x)$ for downstream structured parsing. An illustrative template appears in Figure 2.

11 $$\begin{aligned} \operatorname {Prompt}(x)=\textrm{Task}\ \oplus \ C_x\ \oplus \ x. \end{aligned}$$

where $\oplus$ represents concatenation, $\textrm{Task}$ represents the fixed instruction segment (the red content in Figure 2), and $C_x$ represents the serialized candidate block derived from the retrieved $Top\!-\!K$ communities.

### Constrained inference parsing

The constrained inference parsing module leverages structured control through prompt construction to guide an on-premises LLM (DeepSeek R1) in generating accurate and stable address outputs. This design mitigates hallucination risks associated with free-form generation and enforces a schema-consistent representation aligned with administrative hierarchies^25^. The module takes as input the constrained prompt ($\operatorname {Prompt}(x)$, Fig. 2), incorporating the candidate set and schema constraints from prior stages, and outputs a hierarchical JSON record covering administrative division, street or township, and community.

To ensure compliance with medical data privacy and security requirements, the entire pipeline is deployed on-premises without any external data transfer. Inference is constrained by candidate injection, where the Top-K candidate set ($C_x$) defined in Eq. 11 is embedded in the prompt together with a fixed JSON schema. The prompt requires that the community name be selected from the provided candidates, which prevents the model from introducing address content that is not grounded in ($C_x$).

When the community is not covered by ($C_x$) (out-of-Top-K), the prompt does not provide a correct selectable entry. In this case, the module follows a rejection-first rule encoded in the prompt and returns NULL for all fields rather than forcing an incorrect match. For typographical errors, abbreviations, or partial descriptions, the module relies on the bounded candidate context and the administrative hierarchy encoded in the prompt to support semantic matching when the correct entry appears in ($C_x$). This prompt-explicit abstention pattern reduces unsupported non-null outputs and is consistent with observations in in-context information extraction that large language models tend to over-predict non-null labels when a rejection option is not specified^26^. The corresponding out-of-Top-K behaviors are quantified in Table 9.

## Experimental results and analysis

### Dataset

The dataset was randomly sampled 10,000 records from the institution’s operational database and was minimally normalized prior to annotation in accordance with GB/T 2260-2018. Normalization in-cluded de-identification, removal of bracketed substrings, and unification of full-width and half-width characters. To characterize noise and structural incompleteness observed in practice, an annota-tor-defined taxonomy was applied during labeling. Table 1 reports seven categories. Structurally Valid denotes samples whose five fields are jointly consistent with the national standard. Orthographic Vari-ants covers spelling or character variations that preserve the intended semantics. Address Abbreviation refers to conventional shortenings that can be deterministically resolved to a unique canonical form. Missing Administrative District indicates absent or inconsistent mid-level hierarchy such as the city or district, which yields incomplete administrative context. Non-existent Address comprises entries that cannot be verified against authoritative administrative registers or institutional knowledge bases. Fuzzy or Ambiguous Abbreviation includes shortened forms or aliases with multiple plausible expansions that require contextual disambiguation. Other Errors aggregate residual cases such as malformed numerals, mixed-language tokens, and severe word-order perturbations. This distribution is subsequently used for stratified evaluation and error attribution in order to isolate the impact of hierarchy omissions, abbrevia-tion ambiguity, and orthographic variation on parsing performance.

**Table 1:** Dataset type distribution.

Label	Value
Structurally Valid	7.2%
Orthographic Variants	8.3%
Address Abbreviation	12.4%
Missing Administrative District	48.4%
Non-Existent Address	7%
Fuzzy/Ambiguous Abbreviation	13.2%
Other Errors	3.5%

### Model configuration

The experiments use Python 3.10.16. Modeling and training are implemented with PyTorch 2.6.0+cu124. Four NVIDIA RTX 3060 GPUs with 12 GB memory are used, and the CUDA version is 12.8. The hyperparameters are listed in Table 2.

**Table 2:** Hyperparameter settings.

Parameter	Value
SBERT Embedding	384
GraphSAGE layers	2
Top-*K*	15
LLM	DeepSeek R1 Distill Qwen 32B
LLM temperature	0.8
LLM max length	1024

Evaluation adopts micro and macro accuracy for field-wise extraction. The metrics are defined as:

12 $$\begin{aligned} A_{\text {micro}} = \frac{1}{N}\sum _{i=1}^{N}\prod _{f\in F}{\textbf{1}}\!\left( {\hat{y}}^{\,f}_{i}=y^{f}_{i}\right) , \end{aligned}$$

13 $$\begin{aligned} A_{\text {macro}} = \frac{1}{|F|}\sum _{f\in F}\frac{1}{N}\sum _{i=1}^{N}{\textbf{1}}\!\left( {\hat{y}}^{\,f}_{i}=y^{f}_{i}\right) . \end{aligned}$$

where *N* represents the number of instances, $F=\{\text {city},\text {district},\text {street},\text {community}\}$ represents the set of fields with $|F|=4$, ${\hat{y}}^{\,f}_{i}$ is the predicted label for field *f* in instance *i*, and $y^{f}_{i}$ is the gold label. ${\textbf{1}}(\cdot )$ is the indicator function, which equals 1 if the condition holds and 0 otherwise. $A_{\text {micro}}$ requires all fields of an instance to be correct (joint-field criterion), while $A_{\text {macro}}$ first computes accuracy per field across instances and then averages these accuracies across fields, reducing sensitivity to field-frequency imbalance.

**Training further adopts a contrastive objective with in-batch negatives. The formulation is given as follows.** Let *N* denote the number of training instances. Two query representations are constructed for each instance *i*, including a full address query ${q_i}^{full}$ and a community name query ${q_i}^{name}$,both encoded by Sentence-BERT and projected by a linear layer into the embedding space of dimension *d*. Community node embeddings are produced by GraphSAGE under three graph views, yielding $\{ {q_i}^{full}, {q_i}^{mid}, {q_i}^{comm} \}$ as the positives paired with instance *i*, where in practice the positive index is determined by the community name node mapped from the instance. The InfoNCE loss uses in batch negatives induced by the similarity matrix over all instances within an epoch. For a query set $Q={\{ q_i \}_{i=1}}^N$ and a positive set $P={\{ p_i \}_{i=1}}^N$, the logits are computed as $s_{ij}=<norm(q_i), norm(p_j)>/\gamma$, where $\gamma =0.07$ and $norm(\cdot )$ denotes $\ell _2$ normalization. The supervision assigns label $i=j$ for each $q_i$, which treats $\{p_j\}_{j \ne i}$ as negatives and yields approximately $N-1$ in batch negatives per query. The multi view objective averages three InfoNCE terms over $\{ {q_i}^{full}, {q_i}^{mid}, {q_i}^{comm} \}$ for each query type, and then averages the full address branch and the community name branch.

**Hard negative augmentation**. To expose the model to lexically similar confounders, one hard negative index is assigned to each instance using Sentence-BERT embeddings of community names. Let $e_i$ denote the normalized Sentence-BERT embedding of the community name for instance *i*. The hard negative index is defined as $h(i)={argmax}_{j \ne i}<e_i, e_j>$, where self similarity is excluded by masking the diagonal. The hard negative community embedding is then taken as $n_i=p^{full}_{h(i)}$. During training, a hard negative contrast set is formed by concatenating all positives and all hard negatives, $P^{hard} = [{p_1}^{full}, \dots , {p_N}^{full}, n_1, \dots , n_N]$. The hard negative loss computes logits between ${\{{q_i}^{full}\}^N}_{i=1}$ and $P^{hard}$ with the same temperature $\gamma$ and assigns label $i=j$ for each query ${q_i}^{full}$. The final training loss is ${\mathcal {L}} =\frac{{\mathcal {L}}_\textrm{full}+{\mathcal {L}}_\textrm{name}}{2}+0.5\mathrm {~}{\mathcal {L}}_\textrm{hard}$, which is consistent with the implementation that combines multi view positives with an auxiliary hard negative term.

### Compared models

Levenshtein^27^: A classical string-matching algorithm that measures the minimum number of in-sertions, deletions, and substitutions required to transform one string into another. In this study, it is applied to compute similarity between raw addresses and candidate entries.

Jaro-Winkler^28^: A string similarity metric that assigns higher weights to common prefixes, there-by improving robustness in short-text address matching. It is opted to evaluate pairwise similarity be-tween input and standard addresses.

TF-IDF^29^: A traditional information retrieval baseline that represents addresses as sparse term-frequency vectors weighted by inverse document frequency. Cosine similarity is employed to compute pairwise relevance scores.

BiLSTM-CRF^17^: A sequence-labeling model that combines bidirectional LSTM encoders with a conditional random field layer to capture both contextual semantics and label dependencies. The model is fine-tuned on the address parsing dataset.

BERT-CRF^6^: A pretrained transformer-based encoder (BERT) augmented with a CRF decoding layer. The model leverages contextual embeddings from BERT to segment and label address elements, and is fine-tuned on the same dataset.

Qwen^30^: A LLM baseline. The official API with the full-parameter release of Qwen3 is used. Task-specific instructions are combined with the raw address input in a prompt and input format, and the model outputs standardized address fields through inference.

DeepSeek^13^: Another LLM baseline. The official API with the full-parameter release of DeepSeek R1 is employed. Similar to Qwen, task-specific instructions together with the input address are provided in a prompt and input format to obtain parsing results without additional constraints.

Ali API: A widely used commercial API that provides address standardization services(https://addrp.console.aliyun.com). Raw address inputs are submitted to the API, and the returned results are directly adopted as baseline outputs.

### Experimental results and analysis

Table 3 summarizes the experimental results of the proposed method and all baseline models on the patient address dataset, evaluated in terms of both micro-level accuracy ($A_{\text {micro}}$) and macro-level accuracy ($A_{\text {macro}}$). Several observations can be made.

**Table 3:** Results on datasets.

Model	$A_{\text {micro}}$ (%)	$A_{\text {macro}}$ (%)
AddrKG-LLM (ours)	**73.3**	**80.2**
Levenshtein	35.8	55.4
Jaro-Winkler	42.2	63.9
TF-IDF	49.6	67.1
BiLSTM-CRF	55.0	69.1
BERT-CRF	61.3	71.6
Qwen	40.2	61.6
DeepSeek	37.5	63.3
Ali API	43.8	73.5

Traditional string-similarity methods, including *Levenshtein*, *Jaro-Winkler*, and *TF-IDF*, exhibit limited effectiveness. *Levenshtein* achieves only $35.8\%$ in $A_{\text {micro}}$, reflecting its sensitivity to word-order variations and inability to model semantic relations. *Jaro-Winkler* improves macro-level performance to $63.9\%$ by emphasizing common prefixes, yet still struggles with structural inconsistencies. *TF-IDF* performs relatively better ($A_{\text {micro}}=49.6\%$, $A_{\text {macro}}=67.1\%$) owing to its vector-space representation, but remains constrained by the absence of contextual semantics.

Neural sequence labeling baselines provide notable improvements. *BiLSTM-CRF* attains $A_{\text {micro}}=55.0\%$ and $A_{\text {macro}}=69.1\%$, confirming the benefit of contextual sequence modeling combined with label-dependency constraints. Incorporating pretrained contextual embeddings, *BERT-CRF* further raises accuracy to $61.3\%$ ($A_{\text {micro}}$) and $71.6\%$ ($A_{\text {macro}}$), which underscores the utility of pretrained language models in capturing the semantic and syntactic variability of address expressions.

In contrast, LLM baselines accessed via their official full-parameter APIs, namely *Qwen3* and *DeepSeek R1*, yield suboptimal results despite their strong generative capacity. *Qwen* records $A_{\text {micro}}=40.2\%$ and $A_{\text {macro}}=61.6\%$, while *DeepSeek* achieves $A_{\text {micro}}=37.5\%$ and $A_{\text {macro}}=63.3\%$. The relatively low performance is primarily due to the unconstrained nature of their generation, which frequently introduces extraneous tokens or fails to preserve field consistency, thereby limiting their applicability in structured parsing tasks without additional constraints.

The *Ali API* baseline, representing a commercial solution, achieves competitive macro accuracy ($73.5\%$) but relatively weak micro accuracy ($43.8\%$). This result suggests that while such API can normalize frequent address patterns, it struggles when strict field-level correctness across all four elements is required.

The proposed *AddrKGLLM* consistently surpasses all baselines, achieving $A_{\text {micro}}=73.3\%$ and $A_{\text {macro}}=80.2\%$. Compared with the strongest neural baseline (BERTCRF), the improvement exceeds twelve percentage points in micro accuracy. These gains stem from three critical design elements:

(i) the semantic-constrained retrieval mechanism, which mitigates errors arising from homonymous or incomplete addresses.

(ii) the hierarchy-aware contrastive learning objective, which aligns textual and graph-based representations without supervision.

(iii) the constrained decoding strategy, which restricts the model outputs to candidate sets and enforces JSON schema compliance, thereby reducing hallucination and ensuring structural validity.

Taken together, the results validate the effectiveness of integrating knowledge graph–based structural priors with controlled LLM inference. The proposed approach not only advances parsing accuracy but also enhances robustness and reproducibility under real-world healthcare constraints, demonstrating its potential for large-scale patient address governance.

### Complexity analysis

To further assess the computational characteristics of the proposed framework, Table 4 reports accuracy alongside the average inference time per sentence across different LLM backbones. The results highlight the inherent trade-off between model scale, accuracy, and efficiency.

**Table 4:** Results of *AddrKG-LLM* with different LLM configurations.

Model	$A_{\text {micro}}$ (%)	$A_{\text {macro}}$ (%)	**Time (s/sent.)**
**AddrKG-LLM (DeepSeek R1-32B)**	**73.3**	**80.2**	80.42
AddrKG-LLM (DeepSeek R1-14B)	72.9	79.9	61.35
AddrKG-LLM (DeepSeek R1-7B)	63.4	70.4	**35.31**
AddrKG-LLM (DeepSeek R1-1.5B)	34.1	45.9	52.23
AddrKG-LLM (Qwen3-14B)	66.3	71.2	25.45

The largest configuration, *AddrKG-LLM* (DeepSeek R1-32B), achieves the highest accuracy ($A_{\text {micro}}=73.3\%$, $A_{\text {macro}}=80.2\%$) but requires 80.42 seconds per sentence on average. While this setting yields the strongest parsing performance, its computational overhead renders it less suitable for latency-sensitive applications.

Table 4 indicates a trade-off among scale, accuracy, and latency. *AddrKG-LLM* (DeepSeek R1-14B) maintains near-peak accuracy ($A_{\text {micro}}=72.9\%$, $A_{\text {macro}}=79.9\%$) with 61.35 s/sent and provides a balanced profile. The 7B variant is faster (35.31 s/sent) but reduces accuracy ($A_{\text {micro}}=63.4\%$, $A_{\text {macro}}=70.4\%$), and the 1.5B variant underperforms ($A_{\text {micro}}=34.1\%$, $A_{\text {macro}}=45.9\%$). *Qwen3-14B* presents a different accuracy and latency profile with 25.45 s/sent and moderate accuracy ($A_{\text {micro}}=66.3\%$, $A_{\text {macro}}=71.2\%$), which indicates an architectural effect beyond parameter count.

The higher delay at the highest accuracy setting arises mainly from autoregressive decoding together with long schema-constrained prompts, both of which increase the effective number of generated tokens and the per-step overhead.

Given these characteristics, the current implementation is most suitable for batch-oriented address governance and latency-tolerant on-premises deployments. For interactive scenarios with strict latency budgets, smaller backbones and system-level optimizations are recommended, including prompt and template compaction, tighter Top-*K* candidate sets, tokenizer and KV cache reuse, speculative decoding, parallel retrieval with micro batching, and I/O pipelining. A comprehensive engineering study of these options is left to future work.

### Ablation study

Table 5 summarizes the ablation results of AddrKG-LLM. Excluding GraphSAGE (68.7%/75.5%) diminishes the model’s ability to incorporate hierarchical signals, whereas removing the contrastive learning objective (66.2%/73.4%) weakens semantic–structural alignment. The performance drop becomes more pronounced when both GraphSAGE and CL are removed (63.0%/72.6%), suggesting that structural priors and representation alignment are complementary in enhancing address embeddings. The sharpest decline is observed when prompt design is omitted (45.3%/60.8%). Without constrained prompting, the model frequently generates inconsistent or hallucinated fields, severely undermining end-to-end parsing accuracy. These findings confirm that graph-aware aggregation, self-supervised contrastive learning, and constrained decoding are all indispensable for achieving robust and accurate address parsing.

**Table 5:** Ablation results of *AddrKG-LLM*.

Model	$A_{\text {micro}}$ (%)	$A_{\text {macro}}$ (%)
**AddrKG-LLM**	**73.3**	**80.2**
w/o GraphSAGE	68.7	75.5
w/o CL	66.2	73.4
w/o CL + GraphSAGE	63.0	72.6
w/o Prompt Design	45.3	60.8

To assess robustness across annotator-defined error types, a stratified evaluation was conducted and the results are reported in Table 6. The pattern $A_{\text {micro}} \ge A_{\text {macro}}$ holds across all slices, as expected given that $A_{\text {micro}}$ enforces joint correctness over four fields.

*Address Abbreviation* exhibits the strongest performance, indicating that deterministic normalization combined with candidate-restricted decoding effectively resolves conventional shortenings.

*Missing Administrative District* also performs strongly, reflecting the utility of knowledge-graph structural priors and graph-aware aggregation for recovering absent hierarchical context.

Performance on *Orthographic Variants* is consistent with the effect of light normalization and the hierarchy-aware contrastive objective in absorbing character-level variation while preserving semantics.

For *Non-existent Address*, controlled generation together with a null-fallback policy suppresses spurious completions and improves field-level consistency.

By contrast, *Fuzzy/Ambiguous Abbreviation* remains the most challenging slice because multi-mapping aliases demand stronger contextual disambiguation.

*Structurally Valid* shows closely matched micro and macro accuracy, suggesting alignment between field-wise and joint correctness in this subset, whereas *Other Errors* yields relatively high yet less stable scores due to heterogeneous composition and limited support, and should be interpreted with caution.

In summary, the slice-wise outcomes align with the ablation findings: graph-aware aggregation facilitates hierarchy recovery, the contrastive objective strengthens semantic–structural alignment under orthographic and abbreviation noise, and constrained prompting reduces over-generation in unverifiable cases.

**Table 6:** Per-category accuracy of *AddrKG-LLM* under annotator-defined error types.

AddrKG-LLM	$A_{\text {micro}}$ (%)	$A_{\text {macro}}$ (%)
Structurally Valid	71.4	71.4
Orthographic Variants	73.3	78.3
Address Abbreviation	77.2	85.9
Missing Administrative District	74.7	82.2
Non-Existent Address	76.6	80.8
Fuzzy/Ambiguous Abbreviation	68.7	71.8
Other Errors	80.0	80.0

### Case study

Table 7 reports the effect of varying the depth of GraphSAGE layers in *AddrKG-LLM*. Without graph aggregation (0-layer), performance remains moderate, indicating that semantic-constrained retrieval and controlled LLM inference already provide notable gains, but hierarchical dependencies across administrative divisions are insufficiently captured. Introducing one or two layers of aggregation substantially improves results, with the two-layer configuration achieving the best balance ($A_{\text {micro}}=73.3\%$, $A_{\text {macro}}=80.2\%$). This suggests that limited neighborhood propagation enriches community representations with higher-level geographic context and enhances the model’s capacity to resolve ambiguous or incomplete address mentions.

When the depth increases beyond two layers, accuracy begins to decline. Three layers show noticeable degradation, while four layers cause a sharp drop in both micro and macro accuracy, which can be attributed to over-smoothing: node embedding becomes less discriminative as signals from distant and potentially noisy neighbors dominate. These results confirm that moderate propagation depth is essential for capturing hierarchical structure without introducing excessive noise, and that two-layer GraphSAGE offers the most effective trade-off for this task.

**Table 7:** Results of *AddrKG-LLM* with different GraphSAGE layers.

AddrKG-LLM	$A_{\text {micro}}$ (%)	$A_{\text {macro}}$ (%)
0-layer GraphSAGE	68.7	75.5
1-layer GraphSAGE	72.9	79.9
**2-layer GraphSAGE**	**73.3**	**80.2**
3-layer GraphSAGE	71.1	76.8
4-layer GraphSAGE	28.5	29.0

Table 8 reports the effect of varying the number of retrieved candidates (*K*) in *AddrKG-LLM*. With a small candidate pool (Recall@5), performance remains limited ($A_{\text {micro}}=61.5\%$, $A_{\text {macro}}=67.2\%$), as the correct address is often excluded, restricting the effectiveness of semantic-constrained retrieval and downstream generation. Expanding to Recall@10 substantially improves accuracy ($A_{\text {micro}}=67.5\%$, $A_{\text {macro}}=73.0\%$), indicating that enlarging the retrieval set increases the likelihood of covering the gold address while still maintaining manageable inference overhead.

**Table 8:** Results of *AddrKG-LLM* with different **Top-***K*.

AddrKG-LLM	$A_{\text {micro}}$ (%)	$A_{\text {macro}}$ (%)	**Time (s/sent.)**
Recall@5	61.5	67.2	**63.35**
Recall@10	67.5	73.0	75.46
Recall@15	73.3	80.2	80.42
Recall@20	73.9	80.4	82.25
Recall@30	74.0	81.1	86.29
Recall@50	**74.9**	**82.1**	91.81

Performance continues to improve as *K* increases, with the Recall@15 configuration achieving a strong balance ($A_{\text {micro}}=73.3\%$, $A_{\text {macro}}=80.2\%$). Further expansion to Recall@20, Recall@30, and Recall@50 yields incremental gains, peaking at $A_{\text {macro}}=82.1\%$. However, larger candidate sets also increase inference cost (e.g., 91.81 seconds for Recall@50), reflecting a trade-off between retrieval coverage and computational efficiency. These results confirm that moderate candidate set sizes (e.g., *Recall@15* or *Recall@20*) are sufficient to ensure high recall of the gold address while avoiding excessive computational burden, thereby offering the most effective trade-off for large-scale address parsing.

**Table 9:** Top-K coverage and Out-of-TopK behaviors.

*K*	R@K(name)	R@K(tuple)	Out	Abstain	Force	$Force_{\text {all}}$
5	0.699	0.692	0.301	0.805	0.195	0.0587
10	0.755	0.744	0.245	0.751	0.249	0.0610
20	0.804	0.793	0.196	0.707	0.293	0.0574
30	0.824	0.813	0.176	0.687	0.313	0.0551
50	0.838	0.827	0.162	0.674	0.326	0.0528

Table 9 reports Top-K coverage and out-of-TopK behaviors under candidate-restricted prompting. Here, $Out=1-R@K(name)$ denotes the fraction of queries whose community is not retrieved into the Top-K candidate set. *Abstain* and *Force* are measured on out-of-TopK cases, and ${Force}_{all} = {Out}\times {Force}$ summarizes the overall forced-pick rate.

As *K* increases, both *R*@*K*(*name*) and *R*@*K*(*tuple*) improve, while *Out* decreases from 0.301 at $K=5$ to 0.162 at $K=50$. This trend indicates that larger candidate sets expand the supported region in which the model can infer a correct structured output from the provided candidates, which is particularly important for inputs with typographical noise, abbreviations, or partial descriptions.

For out-of-TopK cases, the module predominantly abstains rather than forcing a match, with *Abstain* remaining higher than *Force* across all *K*. Although *Force* increases with larger *K*, the overall forced-pick rate ${Force}_{all}$ decreases from 0.0587 to 0.0528 because the out-of-TopK fraction shrinks. These results support the rejection-first design encoded in the prompt, which reduces erroneous insertion when the community is not retrievable within the bounded candidate set.

## Conclusion

A joint framework, AddrKG-LLM, was presented for structuring free-text patient addresses by integrating knowledge-graph–enhanced semantic-constrained retrieval, a hierarchy-aware self-supervised contrastive objective, and candidate-restricted on-premises LLM inference with JSON-schema–compliant outputs. The design mitigates hallucination, enforces field-level consistency, and improves controllability by aligning textual and graph representations under explicit administrative constraints. Evaluation on real-world hospital data indicates consistently higher micro/macro accuracy than string-matching, sequence-labeling, and generic LLM baselines, while maintaining a favorable accuracy–efficiency trade-off under resource-constrained settings. Mid-scale backbones and moderate Top-K can-didate pools deliver strong performance with practical latency, supporting large-scale deployment.

All processing and inference are executed locally over de-identified inputs, ensuring compliance with healthcare privacy requirements and facilitating integration into hospital information systems for integration into hospital information systems for privacy-preserving address structuring and validation in batch or latency-tolerant workflows. These properties underscore the framework’s applicability to priva-cy-sensitive environments.

Despite these strengths, several limitations remain. First, inference latency can be substantial when large autoregressive backbones are used with schema-constrained prompts, which restricts suitability for interactive scenarios and favors batch-oriented deployment. Second, the candidate-restricted decoder is inherently bounded by retrieval coverage. When the gold community is not included in the Top K candidate set, the system may abstain by returning null fields, and occasional forced selection can still occur within the candidate list. This behavior implies that end-to-end performance is constrained by retrieval Recall at K. Third, the contrastive objective relies on in-batch negatives and a single nearest-neighbor hard negative per instance, which may not sufficiently represent hierarchy-local confounders such as lexically similar communities within the same district or township, and may be affected by aliasing that introduces ambiguous supervision.

Future work will focus on improving practicality and robustness under real-world constraints. Priority directions include latency reduction through prompt compaction and efficient inference strategies, retrieval enhancement with calibrated open-set rejection to better handle out-of-Top K conditions, and hierarchy-aware hard negative construction that better reflects local ambiguity patterns. Additional evaluation across institutions and temporal shifts will further assess generalization and support long-term maintenance of the address knowledge graph.

## Data Availability

A 10% de-identified sample of the dataset (1,000 address records) used in this study is publicly available at https://github.com/JinzheLi/AddrKG-LLM/tree/main/data. The remaining de-identified records are stored in the secure institutional data warehouse and are not publicly available due to institutional privacy and data-governance policies. Additional data may be obtained from the corresponding author upon reasonable request and with permission from Civil Aviation General Hospital.

## Code availability

The complete source code of AddrKG-LLM is available at the following GitHub repository: https://github.com/JinzheLi/AddrKG-LLM.
